# Factors that influence treatment decisions: A qualitative study of racially and ethnically diverse patients with low‐ and very‐low risk prostate cancer

**DOI:** 10.1002/cam4.5405

**Published:** 2022-11-20

**Authors:** Alice Guan, Janet K. Shim, Laura Allen, Mei‐Chin Kuo, Kathie Lau, Zinnia Loya, James D. Brooks, Peter R. Carroll, Iona Cheng, Benjamin I. Chung, Mindy C. DeRouen, Dominic L. Frosch, Todd Golden, John T. Leppert, Daphne Y. Lichtensztajn, Qian Lu, Debora L. Oh, Weiva Sieh, Michelle Wadhwa, Scarlett L. Gomez, Salma Shariff‐Marco

**Affiliations:** ^1^ Department of Epidemiology & Biostatistics University of California San Francisco California USA; ^2^ Department of Social & Behavioral Sciences University of California San Francisco California USA; ^3^ Department of Urology Stanford University Stanford California USA; ^4^ Department of Urology University of California San Francisco California USA; ^5^ Center for Health Systems Research Sutter Health/Palo Alto Medical Foundation Research Institute Palo Alto California USA; ^6^ Department of Health Disparities Research University of Texas MD‐Anderson Cancer Center Houston Texas USA; ^7^ Department of Population Health Science and Policy Icahn School of Medicine at Mount Sinai New York New York USA

**Keywords:** prostate cancer, psychosocial studies, quality of life, translational research

## Abstract

**Background:**

Factors that influence prostate cancer treatment decisions are complex, multifaceted, and personal, and may vary by race/ethnicity. Although research has been published to quantify factors involved in decision‐making, these studies have been limited to primarily white, and to a lesser extent, Black patients, and quantitative studies are limited for discerning the cultural and contextual processes that shape decision‐making.

**Methods:**

We conducted 43 semi‐structured interviews with a racially and ethnically diverse sample of patients diagnosed with low‐ and very‐low risk prostate cancer who had undergone treatment for their prostate cancer. Interviews were transcribed, independently coded, and analyzed to identify themes salient for decision‐making, with attention to sociocultural differences.

**Results:**

We found racial and ethnic differences in three areas. First, we found differences in how socialized masculinity influenced patient's feelings about different treatment options. Second, we found that for some men, religion and spirituality alleviated anxiety associated with the active surveillance protocol. Finally, for racially and ethnically minoritized patients, we found descriptions of how historic and social experiences within the healthcare system influenced decision‐making.

**Conclusions:**

Our study adds to the current literature by expounding on racial and ethnic differences in the multidimensional, nuanced factors related to decision‐making. Our findings suggest that factors associated with prostate cancer decision‐making can manifest differently across racial and ethnic groups, and provide some guidance for future research.

## Graphical abstract

Factors that influence prostate cancer treatment decisions are complex, multifaceted, and personal, and may vary by race and ethnicity. We conducted 43 semi‐structured interviews with a racially and ethnically diverse sample of patients diagnosed with low‐ and very‐low risk prostate cancer who had undergone treatment for their prostate cancer. We found racial and ethnic differences in three factors that influenced decision‐making: socialized masculinity, religion and spirituality, and historic and social experiences within the healthcare system.

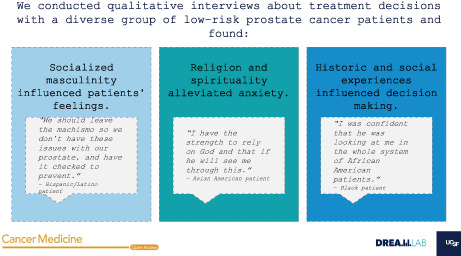

## 1 INTRODUCTION

Since 2018, clinical guidelines have recommended that patients with low‐risk prostate cancer (PCa) consider active surveillance (AS),^1^, ^2^, ^3^ which involves serial prostate specific antigen (PSA) testing, digital rectal examination, and prostate needle biopsy.^4^ With AS, curative therapies are deferred until disease progression is observed,^5^ minimizing potential negative outcomes associated with treatment that can substantially reduce quality of life.^6^, ^7^ Despite recommendations for the use of AS to manage low‐risk PCa,^8^ not all patients receive AS.

Several factors described as important to PCa treatment decisions include trust in physician's recommendations, concerns about side effects of treatment, and family participation in the decision‐making process.^9^, ^10^, ^11^, ^12^, ^13^ Furthermore, factors associated with rejection of AS include fear of disease progression, perception of AS as “doing nothing”,^14^, ^15^, ^16^, ^17^, ^18^, ^19^, ^20^ and unmet information and support needs.^21^, ^22^, ^23^, ^24^, ^25^ Altogether, PCa treatment decision‐making is multidimensional, complex, and highly personal.^16^

Although there are some racial and ethnic differences in recommendations for prostate cancer screening (e.g., the Amerian Cancer Society recommends that Black men and men with a first degree relative with prostate cancer diagnosed at an earlier age seek a clinical encounter at age 45),^26^, ^27^ there are no documented differences in treatment guidelines by race and ethnicity. Despite this, racial and ethnic differences in PCa treatment and decision‐making have been demonstrated.^28^, ^29^ Previous studies have found that Black patients tend to receive less aggressive therapies,^30^, ^31^, ^32^, ^33^ and are presented with fewer treatment options.^34^ One study found that Asian American patients were less likely to report that doctors engage them in treatment decision‐making, but did not find disparities for Hispanic/Latino or Black patients.^35^ Shared decision‐making is essential to helping patients understand personally appropriate options; however, lack of understanding into racial and ethnic considerations can hinder optimal patient‐provider communications. Qualitative research has the potential to elucidate key experiences and insights of importance from the patients' perspectives, and thus, is ideally suited to complement quantitative research in helping us to better understand the process of PCa treatment decision‐making across racial and ethnic groups. To address this question, we analyzed qualitative data from a mixed‐methods study investigating treatment decision‐making in a diverse population of patients diagnosed with low‐ and very‐low risk PCa, with a special focus on reporting differences and similarities in sociocultural factors across racial and ethnic groups.

## 2 METHODS

### 2.1 Study sample

The sample consisted of 43 patients who were interviewed between September 2018 and April 2019. Patients with low‐ and very‐low risk PCa (defined by Gleason score and PSA) were identified through the Greater Bay Area Cancer Registry and were mailed a study introduction, brochure, and response form to indicate availability/refusal to participate. The Greater Bay Area Cancer Registry is a population‐based registry that captures all cases of cancer that are diagnosed or treated in the nine Bay Area counties (Alameda, Contra Costa, Marin, Monterey, San Benito, San Francisco, San Mateo, Santa Clara, and Santa Cruz), as mandated by state law. Interviewers contacted patients by telephone to determine eligibility (defined by age 40–79, PCa diagnosis after January 2016, and not having another cancer diagnosis prior to PCa diagnosis). We interviewed a diverse sample including Black, Chinese, Filipino, Hispanic/Latino, and White patients who completed their initial course of disease management to capture perspectives/reflections of decision‐making. In addition to Chinese and Filipino participants, Asian American participants of other ethnic backgrounds were recruited. In our qualitative sample, AS proportions differed by approximately 26% across racial and ethnic groups and were highest among Black and Hispanic patients and lowest among White and Asian American patients. All participants consented to be in the study, and the study was reviewed and approved by the University of California, San Francisco Institutional Review Board, and the California Protection of Human Subjects.

### 2.2 Data collection

A semi‐structured interview guide was designed using prior research and clinical input to elicit patients' diagnosis stories, relevant factors for decision‐making, and reflections following treatment (Supplemental Materials). For this study, we used professional, bilingual certified field staff with years of experience conducting interviews and focus groups in English, Mandarin/Cantonese, and Spanish, across multiple epidemiologic and qualitative research studies. All interview staff received extensive training and onboarding to ensure competency and consistency between interviews, and were managed by a senior research project manager for quality control. Prior to each interview, patients completed a short demographic survey. Interviews were approximately 60 minutes long and patients received a $60 gift card for their participation.

### 2.3 Data analysis

**FIGURE 1:**
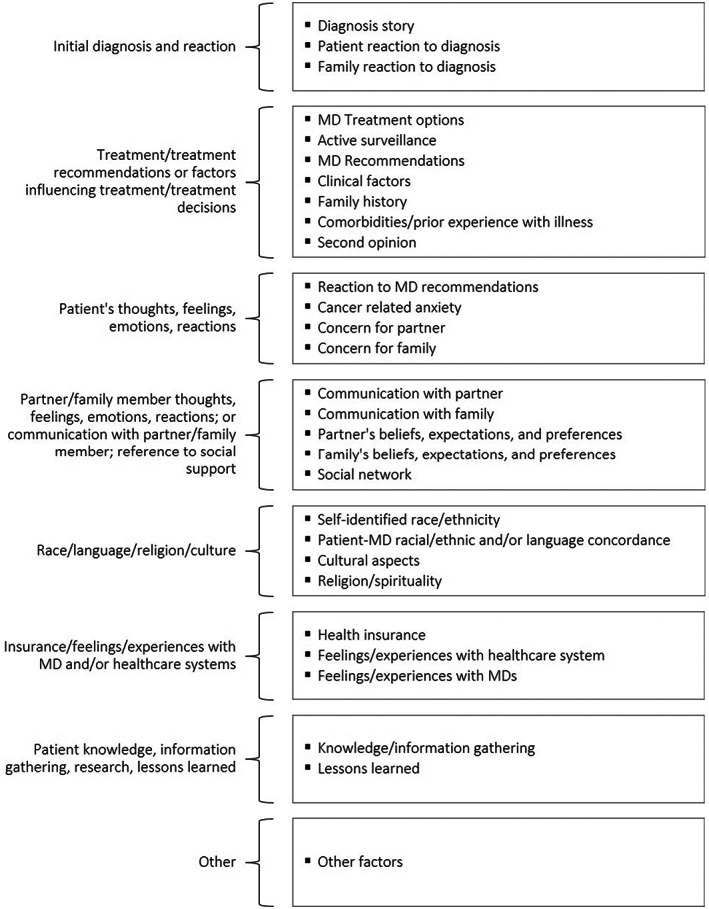
Analytic codebook organized by code themes

Interviews were conducted in English, Spanish, or Cantonese/Mandarin, depending on the participant's preference, then professionally transcribed and translated into English as applicable, reviewed for accuracy, and uploaded to Dedoose, a qualitative data analysis application. Non‐English interviews were simultaneously translated and transcribed by professional translators who participated in the translation/back translation process of study instruments (consents, screeners, invitation letters, semi‐structured interview guides, etc.). A total of 6 Spanish interviews and 3 Mandarin interviews were transcribed and translated in this way. We conducted thematic analysis^36^ as follows: an initial codebook was developed comprised of codes related to the themes we sought to explore in the interviews. Using this initial codebook, three research staff, which included the interviewers, independently coded a test set of three transcripts, then met to compare coding, reconcile differences, and identify new codes that emerged inductively from the interview data. Three rounds of independent coding and discussion resulted in a final set of 29 codes (Figure 1), which were used to analyze the full set of transcripts across racial and ethnic groups. Codes relevant to sociocultural and contextual factors influencing treatment decision‐making (e.g., religion/spirituality, cultural aspects, experiences with healthcare) were reviewed separately for each racial and ethnic group and summarized for comparison across groups.

## 3 RESULTS

### 3.1 Sample characteristics

Of the 43 patients, 30.2% identified as Asian American (5 Chinese, 3 Filipino, and 5 Other Asian American, including Asian Indian, Japanese, South Asian, Vietnamese, and one participant who did not specify their ethnic group), 23.2% as Black, 23.2% as Hispanic/Latino, and 23.2% as White (Table 1). Mean age at diagnosis was 61.2 years old. Most patients were born in the U.S. (62.8%), currently married (79.1%), completed college or more (53.5%), were currently employed (55.8%), had household income of $100,000 (51.2%), and had private health insurance (60.5%). Less than half (44.2%) of the sample opted for active surveillance to manage their PCa.

**TABLE 1:** Sample characteristics (*N* = 43)

Characteristic	N (%) or Mean, SD
Race Ethnicity	
Chinese American	5 (11.6%)
Filipino or other Asian American^a^	8 (18.6%)
Black	10 (23.2%)
Hispanic/Latino	10 (23.2%)
White	10 (23.2%)
Age at Diagnosis	61.2, 7.7
Born in the United States	27 (62.8%)
Marital status	
Currently married or living with a partner as married	34 (79.1%)
Never married, separated, or divorced	9 (20.9%)
Highest level of education completed	
High school/GED or less	7 (16.3%)
Some college	13 (30.2%)
College graduate	11 (25.6%)
Post‐college graduate	12 (27.9%)
Employment Status	
Employed	24 (55.8%)
Unemployed (includes welfare and disability) or self‐employed	5 (11.6%)
Retired	14 (32.6%)
Household size	2.7, 1.4
Total household income	
Less than $100,000	15 (34.9%)
$100,000 to $149,999	7 (16.3%)
$150,000 or more	16 (30.2%)
Do not know/Refused	5 (11.6%)
Health Insurance	
Medi‐Cal or Medicare	10 (23.2%)
Medicare and other (including Medi‐Cal, Private Insurance, and VA)	7 (16.3%)
Private Insurance	26 (60.5%)
Treatment received	
Active surveillance	19 (44.2%)
Active treatment (e.g., radiation, surgery)	24 (55.8%)

^a^ 3 patients self‐identified as Filipino American and 5 self‐identified as another Asian American group. Survey options for Asian American groups included: Chinese, Filipinos, and Other Asians. Specific Asian American subgroups specified for those who selected “other Asian American” included Japanese, South Asian, and Vietnamese.

### 3.2 Qualitative findings

We identified three novel themes related to sociocultural factors influencing treatmentdecision‐making. Specifically, we found racial and ethnic differences in (1) socialized expectations around gender and sexual functioning, (2) religion and spirituality, and (3) provided communication and healthcare experiences in treatment decision‐making. We present example excerpts in each of the themes below, which are illustrative of experiences that were described within each of the racial/ethnic groups.

### 3.3 Socialized expectations surrounding gender and sexual functioning

The first theme we identified related to socialized expectations or norms around gender and sexual functioning. These norms manifested in several distinct ways: as perceptions of masculinity as related to the prostate; the role of a man as a romantic partner or in a family; and also the importance of sexual functioning. Social expectations around gender and sexual functioning were not as influential for White patients with PCa. When asked whether relationship with their partner was an important factor for treatment decisions, most White patients mentioned their autonomy. For example, one patient told us that his partner “was supportive” but that “my relationship … had nothing to do with my prostate decisions.” (White participant)

In contrast, several raically and ethnically minoritized patients described the impact of socialized expectations of masculinity, particularly around sexual intimacy, on their treatment decisions. They described sexual function as important and relevant to their role in intimate relationships, which ultimately influenced their treatment decisions.

As a loving partner, you are supposed to have sexual relationship, so if one of the cons would be that wouldn't have any… that was hard to grasp… It was very hard for me to have the idea that I will not be able to perform as I did before or to lose the intimacy. (Japanese American participant)

It's one of the parts that patients fear losing, the ability to be intimate with your partner. If life is hard, can you imagine not being able to have relations with your partner? (Hispanic/Latino participant)

With my partner … [it's] really hard on her because the intimacy is not there … I can do without but I am really, really concerned about her. (Black participant)

Two Black patients described the impact that socially conditioned concept of masculinity had on their decision‐making:

Treatment options… still feel a little draconian to me, frankly… Most of them are going to impair us in some way that leaves us slightly humiliated…. This society puts so much into manhood in a particular way, so that if you haven't done the work of realizing that your manhood is not attached to whether you are incontinent, or whether you can get an erection… then your treatment options are going to be influenced by your fear of losing these things that we have associated with manhood … The treatment options do put you right in the face of that kind of fear, that I'm going to no longer be the American cowboy that I've been socialized to be. (Black participant)

I think Black patients, as a whole, move slower on this… almost everybody knows about this incontinence and the loss of erections, which scares the heck out of them … I know it's a biggie among the Black patients that I know, that loss of their erection. (Black participant)

For both these patients, even though they understood that “fear” and “humiliation” around potential erectile dysfunction stemmed from gendered expectations about sexuality, these perceptions still played a role in their own decision‐making and those of other Black patients they knew.

For Hispanic/Latino patients, the concept of masculinity emerged primarily when describing barriers to screening, rather than factors related to decision‐making. Specifically, one of the Hispanic/Latino patients implicated machismo as a factor that hindered their ability to make timely decisions about their medical care.

We should leave the machismo so we don't have these issues with our prostate, and have it checked to prevent. If I had been doing my exams regularly, perhaps I wouldn't have had these issues with my prostate. We need to leave the machismo, at work a bunch of comments were made, made fun of (laughs). I didn't care because it's my life. (Hispanic/Latino participant)

While not directly related to decision‐making, his insight has potential implications for ways in which socialized masculinity in the Hispanic/Latino community could influence medical decision‐making more broadly.

### 3.4 Religious and spiritual considerations

While mentioned less predominantly relative to the other themes, we observed differences in participants' descriptions of the role of religion and spirituality in the treatment decision‐making process by race and ethnicity. Among White participants, only one individual mentioned the importance of religion when asked about this factor. In contrast, more non‐White participants described varying ways in which religion and spirituality were significant for their decision‐making; for example, several shared that their faith helped them accept the unpredictability of cancer treatments and come to terms with health outcomes beyond their control.

I'm at peace in the sense that whatever happens to my body, I have the strength to rely on really on God and that if he will see me through this… I'm ready to go. It's not a big issue. If it did come back aggressive cancer, then I'll address it. (Other Asian American participant)

I decided to live peacefully and work and my family and God and see what happens ahead. (Hispanic/Latino participant)

I have a Christian faith, prayed and sought the face of God and see what He wants me to do and got on the AS… I needed to weigh the options. (Black participant)

These quotes are examples of participants' descriptions of the importance of religion in providing confidence and trust in treatment decisions. For both, faith in a higher power alleviated the anxiety associated with their treatment decision, including allowing them to accept the results of ongoing AS protocols. Although qualitative studies are not meant for making inferences regarding quantitative associations, across our small sample, we did note a higher proportion of Black patients reporting on the relevance of religiosity and spirituality, followed by Hispanic/Latino and Asian patients. The relative importance of this factors should be further evaluated in quantitative studies.

### 3.5 Racial/ethnic differences in communication and healthcare experiences

We observed heterogeneity in the experiences that patients had with their healthcare providers and institutions. Across all racial and ethnic groups, patients expressed faith in their physician's training and affiliation with reputable institutions. Overall, there were patients within all racial and ethnic groups who described positive experiences with their healthcare providers during the treatment decision‐making process. Several Black and Hispanic/Latino patients additionally expressed how past (self/friend/family's) healthcare experiences influenced their willingness to trust treatment recommendations.

I had a lot of trust in this doctor. He worked with my son, so I'd known him before this …. I had a lot of confidence in him and that he knew what he was doing and what he was talking about. (Black participant)

I have [name of insurer], and they'ave always been really good with the family … Always taking care of us. Had really good doctor system … I just [knew] that I was going to be taken care of really well. (Hispanic/Latino participant)

Patients across most racial anethnic groups described positive experiences and trusted the health care system. However, one Black patient articulated a level of skepticism and mistrust about the healthcare system and allopathic medicine generally, expressing an aversion to medical intervention.

I'm fairly skeptical of the healthcare system at large, so I'm sure that factors into any decision … I want you minimally involved in my body … If something dire is happening or likely to happen, or something that Western medicine can help quell quickly, then I'm for it…This felt like this is some sort of slow moving, slow growing thing. So, no, I don't need you to bring your techniques or technology, your medicines, into this right now. (Black participant)

Another Black patient additionally acknowledged that the way in which his beliefs about the fairness of the healthcare system shapes his treatment decision was context‐specific.

If you put me back out in that rural area I grew up in, this would've been a very hard decision to make because I wouldn't have had these beliefs in honesty of the system. Probably if you'd put me in the south, being a Black man, it would change because I'd have a hard time probably rightly or wrongly, that enters into minorities' minds. (Black participant)

Two patients described the importance of racial and ethnic concordance with their healthcare providers in facilitating communication and understanding.

He [the urologist] is knowledgeable, he is African American… I was confident that how he was looking at me in the whole system of African American patients and what he was recommending, it felt reasonable to me. And [it] wasn't in the case of many African American patients who have a faster growing cancer in their bodies. So, I could hear his recommendations and realize that they were reasonable, even with wanting it out of my body. (Black participant)

A few additional themes emerged from those who perceived their racial and ethnic background to be influential. Some Asian American patients described stigma surrounding discussion of PCa. These patients alluded to secrecy in discussing their prostate with family members, implying a taboo with discussing this body part. While describing these feelings of silent suffering, these patients also expressed a desire to talk about their PCa and a wish for more open communication around this topic.

I know that colon cancer seems to be, especially high risk with both sides of my family. But the prostate, patients don't talk about that…. If the medical practitioner orthe medical facility is aware there is a support group somewhere in the community, it would be a great idea… because patients, you know, don't… naturally talk. (Japanese American participant)

[In China,] when they talked about the reproduction system and things like that, you just read it on your own and that was it… [We] don't talk about it too much… There are advantages after talking/discussion, that is–one of which is that it would become clear to you if you didn't know before. You can deal with it properly. You can exchange ideas with each other and learn from… not knowing to‐‐gradually face it more properly. (Chinese American participant)

Patients also described linguistic limitations as a factor that influenced their ability to receive relevant information to inform their treatment decisions, but much of this was dependent on their interactions with their clinicians.

[The doctor] explained the results and the problem of the tumor found in my body in details. He wrote down those numbers because my English is not very well. He also laid out pictures. Told me where that was located on the prostate, etc., and numbers. He spent a long time talking. He also asked for my opinions. (Chinese American participant)

The doctor tried to explain everything with my little English. He was very honest with me, in that aspect, there wasn't like there wasn't any mercy, tactful, no time to prepare, he simply said what it was “you have cancer, you are an age where you can do something because it's in a stage that's not aggressive”. At that time, it helped keep calm. Like I said before, I didn't have anyone to talk with in those terms, or to give me another option. (Hispanic/Latino participant)

These contrasting experiences from patients who spoke limited English suggest that there is variability in the strategies that providers have utilized to communicate across language barriers. While some patients experienced brief, albeit perceived to be straightforward, explanations and encounters with their clinician as a result of their limited English proficiency, others experienced a more meaningful dialog when the physician was willing to incorporate complementary communication methods (e.g., written and visual) into their conversation about diagnosis and treatment options.

## 4 DISCUSSION

While previous qualitative work examining factors related to PCa have elucidated important influences on treatment decision‐making, to our knowledge, this is the first qualitative study to describe racial and ethnic and sociocultural factors associated with decision‐making for patients with low‐ and very‐low risk PCa. Although the factors presented in this study were not the only ones that were described by participants, we focused our findings on describing novel themes across racial and ethnic groups.

Our findings suggest that differences in norms around masculinity could potentially influence an patient's PCa treatment decisions. These norms and expectations manifested in several distinct ways, including perceptions of masculinity that related to the prostate, the role of men as romantic partners or within a family, and the importance of sexual functioning and intimacy. Specifically, racially and ethnically minoritized patients mentioned social norms about masculinity related to erectile and sexual function as well as their roles in romantic and family relationships. While previous quantitative studies have described racial and ethnic differences in the perception of masculinity,^37^ none to date have described how these differences manifest in PCa decision‐making. Future studies should investigate the ways in which norms of masculinity intersect with race and ethnicity and management of cancer.

We found that some patients viewed religious/spiritual beliefs as influential for PCa treatment decision‐making. For this study, we adopted a broad definition of religion, spirituality, and spiritual beliefs, as encompassing connectedness to an organized religion or spiritual being. Prior studies among Black people^38^, ^39^, ^40^ and aggregated racial minoritized groups^41^ suggest a link between religious/spiritual beliefs and cancer decision‐making. Additionally, one study with a primarily White sample found that use of religion/spirituality to cope with PCa differed by baseline levels of religiosity.^42^ Prior research on Hispanic and Asian American patients is scant, but our findings contribute to the the understanding of how sociocultural factors can influence treatment decision‐making in these groups. Future studies should evaluate quantitative impact of religiosity and spirituality across racial and ethnic groups.^43^

Our findings support existing evidence that racially and ethnically minoritized patients experience difficulties in their medical encounters, likely due to bias among healthcare providers and staff, or medical mistrust from prior experiences with the healthcare system. One Black patient mentioned the context‐specific nature of his trust in the healthcare system, specifically pointing to his perception of healthcare in rural America versus in his present geographic context. Our Bay Area sample consisted of patients with higher educational attainment and income compared to the national average. Thus, this particular patient's acknowledgment of geographic context, coupled with his racial and ethnic background, points to a potential intersection of race and ethnicity, socioeconomic status, and geography on healthcare experiences. In fact, the confluence of these three factors has been found to be influential for adverse health outcomes in the Black community.^44^ Additionally, though very limited research has been done on Asian Americans' experiences with PCa, our findings regarding the stigma surrounding cancer and difficulty discussing these issues are consistent with prior studies exploring Chinese American immigrants experiences with breast cancer.^45^ Second, as mentioned by both Black and Asian American patients in this study, physician‐patient racial and ethnic concordance provided confidence in patients's bc2021, Black representation in the U.S. urology workforce only made up approximately 2% of the urologists in the nation compared with approximately 14% of the U.S. population – thus, medical schools and hospitals should implement programs designed to diversify the urologist workforce.^46^, ^47^ In addition, medical education must strive toward including training on how social, historical, and political structures influence health and produce inequality.^48^, ^49^, ^50^

There are several limitations to this study. Because patients in this study had already completed the decision‐making process, our findings may not capture all factors that participants may have considered while they were in the process of making decisions. However, given interviews were conducted shortly after participants decided on their first course of disease management, our findings are likely to accurately capture the experiences of these men. Additionally, many of the norms and expectations regarding masculinity and the role of a man within family systems can differ based on sexual orientation, gender identity, and relationship preferences. Since we did not have this data in our study, we were unable to make inferences regarding this point. However, this will be an important topic for future research, as the number of sexual and gender minorities in the U.S. continues to rise. Finally, our study comprised patients who had a higher level of educational attainment and socioeconomic status compared to other regions of the country. While the complexities in masculinity, religion, and healthcare with relation to treatment decision‐making illustrated in this study capture the experiences of patients with these privileges, future studies are warranted to see how these perspectives differ in other areas.

## 5 CONCLUSION

In summary, our study provides insights into racial and ethnic and sociocultural factors associated with decision‐making among a diverse sample of patients. We uncovered several novel themes related to patients's decision‐making which could help to personalize clinical communications across racial and ethnic groups and inform directions for future research. Though characteristics of patients in other regions may differ, the goal of qualitative research is to develop a more nuanced understanding of beliefs and perceptions, rather than generalize to larger populations. Thus, this study provides useful insights into potential gaps in our understanding of the complex contributions to and ways in which patients facing a low‐ or very‐low risk PCa diagnosis grapple with decisions on whether and how to treat, and how to continue to live with cancer.

## AUTHOR CONTRIBUTIONS

**Alice Guan:** Formal analysis (lead); writing – original draft (lead); writing – review and editing (equal). **Janet Shim:** Conceptualization (lead); formal analysis (lead); methodology (lead); writing – original draft (equal); writing – review and editing (equal). **Laura Allen:** Data curation (equal); formal analysis (equal); project administration (equal); supervision (equal); writing – review and editing (equal). **Mei‐Chin Kuo:** Data curation (equal); formal analysis (equal); writing – review and editing (equal). **Kathie Lau:** Data curation (equal); formal analysis (equal); writing – review and editing (equal). **Zinnia Loya:** Data curation (equal); formal analysis (equal); writing – review and editing (equal). **James D Brooks:** Writing – review and editing (equal). **Peter Carroll:** Writing – review and editing (equal). **Iona Cheng:** Writing – review and editing (equal). **Benjamin Chung:** Writing – review and editing (equal). **Mindy C. DeRouen:** Writing – review and editing (equal). **Dominick L Frosch:** Writing – review and editing (equal). **Todd Golden:** Data curation (equal); formal analysis (equal); writing – review and editing (equal). **John T Leppert:** Writing – review and editing (equal). **Daphne Y Lichtensztajn:** Writing – review and editing (equal). **Qian Lu:** Writing – review and editing (equal). **Debora Lee Oh:** Writing – review and editing (equal). **Weiva Sieh:** Writing – review and editing (equal). **Michelle Wadhwa:** Writing – review and editing (equal). **Scarlett Lin Gomez:** Conceptualization (lead); formal analysis (lead); funding acquisition (equal); methodology (lead); writing – original draft (equal); writing – review and editing (equal). **Salma Shariff‐Marco:** Conceptualization (lead); formal analysis (lead); methodology (lead); writing – original draft (equal); writing – review and editing (equal).

## ETHICS STATEMENT

This study protocol was approved by the Institutional Review Boards at the University of California, San Francisco, and California Protection of Human Subjects at the California Department of Public Health.

## Supporting information


Data S1

## Data Availability

The data that support the findings of this study are available on reasonable request from the principal investigator of the study, SLG. The data are not publicly available as these contain information that could compromise the privacy of research participants.
