# Supplementary material for: Factors that influence treatment decisions: A qualitative study of racially and ethnically diverse patients with low‐ and very‐low risk prostate cancer
Source: Cancer Med. 2022 Nov 20;12(5):6307–17. doi: 10.1002/cam4.5405 (PMC10028041; doi:10.1002/cam4.5405)
Supplement: Supplementary file 1 — Data S1 [file CAM4-12-6307-s001.docx]

**SUPPLEMENTAL MATERIALS**

The following is the topic guide that was used to conduct semi-structured interviews with patients with low- and very-low risk prostate cancer.

- I would like to start by having you describe the “story” of your prostate cancer experience. It would be great if you could start with how you found out that you had prostate cancer.
- As a [specify race or ethnicity] man, what does it mean to you to have prostate cancer?
- What were your thoughts and feelings when you discovered that you have prostate cancer?
- How did your family react to your diagnosis?
- Tell me about the discussion you had with your doctor about your treatment options.
- Did your doctor tell you about different types of prostate cancer?
- Do you remember what he/she said about your prostate cancer?
- Did any of your doctors recommend a particular treatment option?
  - If yes, did you agree with the recommendation?
  - If no, did you feel comfortable expressing disagreement with the recommendation?
  - If not comfortable, what got in the way?
  - If AS not mentioned: You have not brought up AS during the discussion. What, if anything, did your doctor have to say about AS for you? *Probe about pros and cons*.

*If participant asks, “what is active surveillance?”:* Because prostate cancer often grows very slowly, some men might hear from their doctor that they may never need treatment for their prostate cancer. Instead, their doctors may recommend an approach known as active surveillance or sometimes called watchful waiting, observation, or expectant management. Active surveillance means your doctor would monitor you closely by seeing you regularly at office visits and getting tests, but not treating your cancer otherwise. Did your doctor talk about this approach for you?

- What things were important to you when thinking about treatment? Or, please describe how your prostate treatment decision was made.

What personal factors led to the decision about the treatment you received?

- Family history
- Prior experience with illness
- Prior experience with health care system
- Your personal religious beliefs
- Your heritage and family background

What relationship factors led to the decision about the treatment you received?

- Your relationship with your intimate partner
- Your partner’s beliefs about prostate cancer
- Your partner’s expectation about treatment outcomes
- The way you discuss important things with your partner
- Your partner’s concerns about you
- Did your partner have other preferences for your prostate cancer treatment? [If YES] What were they/can you explain?

What health care factors led to the decision about the treatment you received?

- Your relationship with your doctor
- Your medical insurance
- What you know about prostate cancer
- Your beliefs in honesty and fairness of the health care system
- Your trust in your doctors
- Any lessons learned you would like to share with others who might be trying to decide about treatment for their prostate cancer, including active surveillance?
- Do you have other thoughts that we have not covered?
